# Enhanced Remdesivir Analogues to Target SARS-CoV-2

**DOI:** 10.3390/molecules28062616

**Published:** 2023-03-13

**Authors:** Ryuichi Majima, Tiffany C. Edwards, Christine D. Dreis, Robert J. Geraghty, Laurent F. Bonnac

**Affiliations:** Center for Drug Design, College of Pharmacy, University of Minnesota, Minneapolis, MN 55455, USA

**Keywords:** C-nucleoside, antiviral, SARS-CoV-2, phosphate prodrug

## Abstract

We report the short synthesis of novel *C*-nucleoside Remdesivir analogues, their cytotoxicity and an in vitro evaluation against SARS-CoV-2 (CoV2). The described compounds are nucleoside analogues bearing a nitrogen heterocycle as purine analogues. The hybrid structures described herein are designed to enhance the anti-CoV2 activity of Remdesivir. The compounds were evaluated for their cytotoxicity and their anti-CoV2 effect. We discuss the impact of combining both sugar and base modifications on the biological activities of these compounds, their lack of cytotoxicity and their antiviral efficacy.

## 1. Introduction

**Figure 1:**
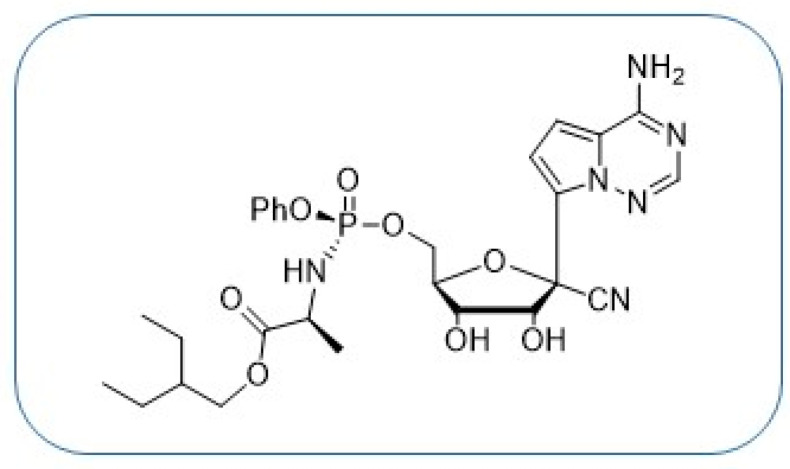
Remdesivir, long synthesis, poor PK, moderate antiviral activity.

Despite the rapid development and approval of safe and efficient vaccines against SARS-CoV-2 (CoV2), the causative agent of the ongoing Coronavirus Disease 2019 (COVID19) pandemic, millions of people have died from COVID19. Vaccine hesitancy, the emergence of novel variants and the scarcity of efficient, orally available drugs to treat severe CoV2 infections makes anti-CoV2 drug research critical to face the COVID19 pandemic and other possible viral threats. In that regard, Remdesivir (Figure 1) is an approved antiviral drug for the treatment of CoV2 infections in many countries. However, Remdesivir must be administered intravenously due to its poor pharmacokinetics, and it only leads to moderate benefits [1], underlining the critical need for more effective alternatives.

**Figure 2:**
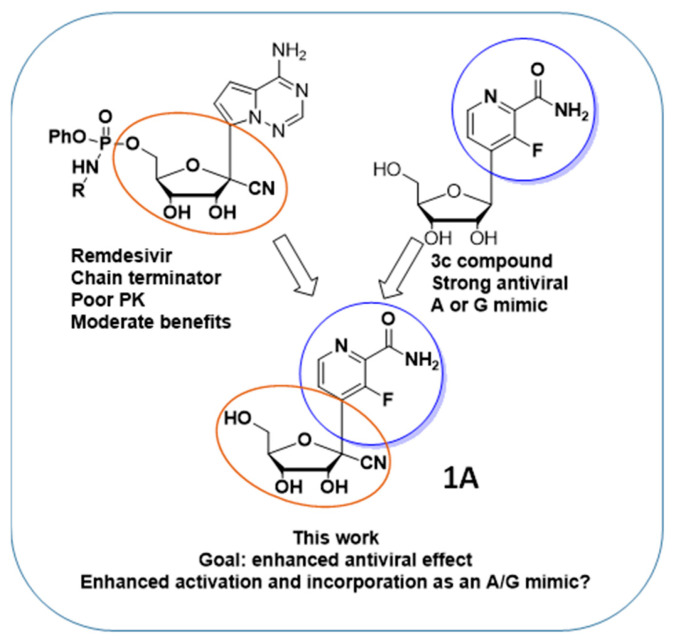
Enhanced Remdesivir analogue.

Remdesivir is active against several RNA viruses, including coronaviruses and Ebola virus [2]. Remdesivir is an adenosine analogue, a *C*-nucleoside with a 5′-phosphate prodrug and a cyano group at the 1′ position of the carbohydrate moiety. In infected cells, Remdesivir is converted to the corresponding triphosphate form, which acts as a potent inhibitor of the viral RNA-dependent RNA polymerase (RdRP) via a mechanism of delayed chain termination, 3 to 5 nucleotides after its incorporation into the viral genome [3,4,5]. Remdesivir features a phosphate prodrug to bypass the often inefficient first phosphorylation of nucleoside analogues [6,7]. The *C*-nucleoside structure of Remdesivir, with a carbon-carbon bond between the base and the sugar (compared to the nitrogen-carbon bond in natural nucleosides), confers an increased chemical and enzymatic stability to Remdesivir. Synthetically, Remdesivir requires many chemical steps to be prepared [8], making a large-scale synthesis and availability challenging. The difficult synthesis, poor pharmacokinetics and moderate activity of Remdesivir makes the search for more active alternatives particularly relevant. In this paper, we describe the short synthesis of a hybrid structure, Remdesivir/3c analogues (**1A**, Figure 2), with the potential for an enhanced bioactivation and incorporation into viral genetic material, and its evaluation against CoV2.

Because of the long chemical synthesis, poor pharmacokinetics and moderate anti-CoV2 activity of Remdesivir, we propose the short synthesis of Remdesivir analogues such as compound (**1A**, Figure 2), with the potential for an enhanced activation and antiviral effect. The rationale for the design of the hybrid structure (**1A**, Figure 2) is as follows: The nucleoside analogue “**3c**”, described by Wang et al. [9], is a *C*-nucleoside that displays a strong antiviral activity (EC_50_ = 1.9 µM, CC_50_ > 400 µM) against influenza virus. The antiviral mechanism of action of **3c** differs from that of Remdesivir. In cells, **3c** is converted to its corresponding triphosphate form and incorporated by the viral RdRP into the growing viral genome as an A or G mimic, leading to the alteration of the viral genome (lethal mutagenesis) and antiviral effect. Interestingly, **3c** is able to mimic A or G thanks to its rotatable amido group. We hypothesized that a Remdesivir/3c hybrid structure would benefit from the different characteristics of each molecule. We designed the hybrid structure (**1A**, Figure 2) containing the Remdesivir ribose modification to obtain an antiviral effect via delayed chain termination, yet combined it with the base of **3c** in order for it to benefit from the amido group mimicking A or G. Consequently, our hybrid structure could possibly benefit from: (1) an increased activation by A or G kinases to its active triphosphate form; and (2) an increased incorporation by the viral RdRP of the active form as an ATP or GTP mimic into the viral genome, therefore leading to an increased antiviral effect via delayed chain termination.

## 2. Results

**Figure 3:**
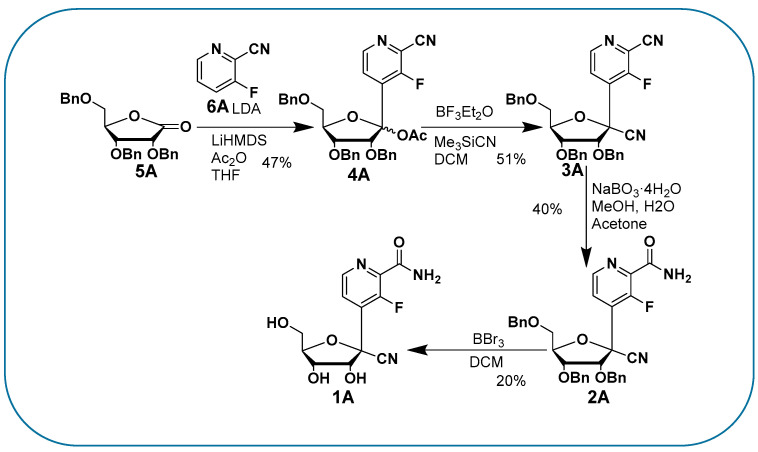
Synthesis of the analogue.

With the reaction described in Figure 3, we proceeded to access our target Remdesivir analogue (**1A**) in four steps, an improvement compared to the long synthesis required for Remdesivir. In the first step, the aromatic moiety (**6A**) is treated with Lithium Diisopropyl.

**Figure 4:**
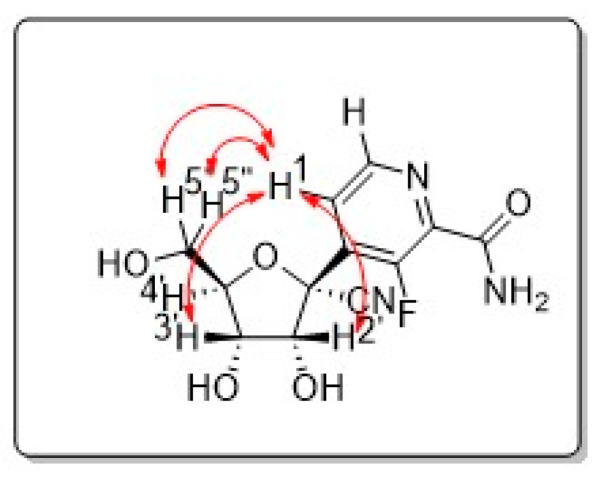
^1^H-^1^H NOESY correlations.

**Figure 5:**
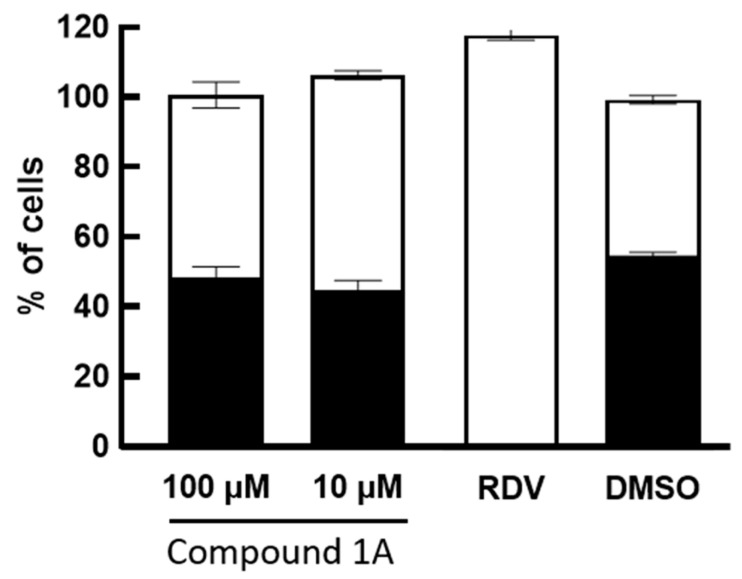
Antiviral activity assay. Percent (%) total cells normalized to the DMSO-treated control are shown as white bars. % infected cells are shown as black bars. Means and standard deviations of triplicate wells are shown. No antiviral effect was detected at any doses tested for compound **1A**. Remdesivir (RDV 2 mM) was used as positive control and resulted in almost complete inhibition of infection.

**Figure 6:**
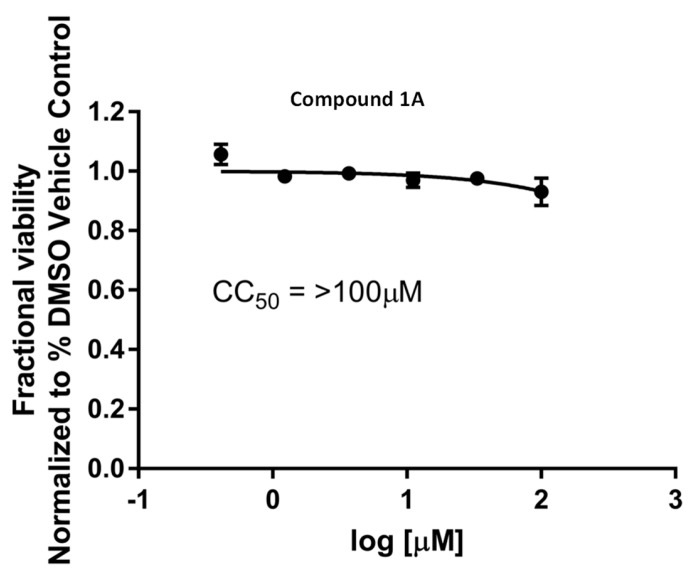
Cytotoxicity measurement.

Amide (LDA) is used to access the organolithiated form of (**6A**), which adds nicely to the carbonyl of the ribonolactone (**5A**). The resulting 1′ hydroxyl is then acetylated in one pot with acetic anhydride to obtain intermediate (**4A**) with a 47% yield. The regioselectivity of the base addition was confirmed by ^19^F NMR and a lack of coupling with protons, which is consistent with the literature [9]. We then proceeded with the addition of the cyano group at 1′ by treating compound (**4A**) with a Lewis acid (Boron trifluoride diethyletherate) in order to access the oxonium intermediate that reacts with trimethylsilylcyanide (use with caution), so as to obtain the beta anomer *C*-nucleoside (**3A**) with a 51% yield [10,11,12]. The desired diastereoselectivity of the reaction, favoring the formation of the beta anomer, is confirmed by ^1^H-^1^H NOESY NMR. The next step, from compound (**3A**) to (**2A**), involved the hydrolysis of the aromatic cyano group to an amido group; albeit apparently simple, it was challenging due to the presence of another cyano group at 1′. A typical hydrolysis of cyano groups to amido groups using potassium trimethylsilanolate (a synthetic equivalent of “O^2−^”) and water [13] failed to provide any selectivity. However, when we used a mild oxidant such as sodium perborate in water and methanol [14], we were pleased to obtain a regioselective hydrolysis of the aromatic cyano group to the amido group without alteration of the 1′ group, obtaining compound (**2A**) with a 40% yield. We then proceeded with the deprotection of the benzyl groups with boron tribromide in dichloromethane, a typical method to deprotect the benzyl groups of *C*-nucleosides, to obtain compound (**1A**) with a 20% yield. It remains unclear to us why the yield was low, as it can be as high as 94%, as we have previously published [15] for similar structures and a similar method. The presence of the cyanide group at 1′ and a difficult purification may explain the low yield. We confirmed compound (**1A**) as a beta anomer by 2D proton-proton NMR with the absence of a correlation between the 4′ proton and the aromatic protons, while correlations were observed between 2′, 3′, 5′ protons and the aromatic protons (Figure 4). We underline that the described reactions and yields are not optimized, since our goal was to obtain compound (**1A**) for an antiviral evaluation as proof of concept. Compound **1A** was evaluated for its antiviral activity (Figure 5) and cytotoxicity (Figure 6).

## 3. Discussion

In order to improve the inefficient, multi-step synthesis, poor pharmacokinetics and moderate antiviral activity of Remdesivir, we proceeded with the generation of hybrid nucleoside analogues. We synthesized compound **1A** (Figure 3), an analogue of Remdesivir, in a short four-step synthesis. The rationale for the synthesis of this compound was to modify the base, so that it could benefit from an increased activation and incorporation into the viral genome by mimicking adenosine and guanosine and behaving as a chain terminator. Compound (**1A**) was evaluated for its cytotoxicity and for its antiviral activity against SARS-CoV-2, as described previously [16]. Compound (**1A**) did not display any antiviral activity against SARS-CoV-2 (Figure 5) and completely lacked cytotoxicity, even at high doses (Figure 6). SARS-CoV-2 can recognize and remove certain non-natural nucleosides thanks to the exoribonuclease activity of auxiliary protein NSP14 [17], which may be the case for our analogues. However, the complete lack of cytotoxicity, even at high doses, may point toward a problem in the conversion of our analogue to its active triphosphate form. The first phosphorylation is often the rate-limiting step. A possibility is to add a monophosphate prodrug, which has been developed to bypass the first phosphorylation bottleneck and increase antiviral nucleoside biological activities [18,19,20]. However, the second or third phosphorylation can also be an issue. In our case, the described combination of modifications of both sugar and base may preclude any activity. Further work is currently underway to limit the impact of modifications on the base while retaining a potentially enhanced antiviral activity via chain termination.

## 4. Materials and Methods

The reagents used to make the growth and infection medias were purchased from the following sources. Dulbecco’s modified Eagle’s medium (DMEM)DMEM high-glucose, fetal bovine serum (FBS) FBS, Streptomycin/Penicillin, HEPES, Non-Essential Amino Acids (NEAA), Sodium Pyruvate, GlutaMAX, Puromycin, Trypsin and PBS were purchased from ThermoFisher (Waltham, MA, USA). Blasticidin and Plasmocin were purchased from InvivoGen (San Diego, CA, USA). Remdesivir was purchased from MedKoo Biosciences (Morrisville, NC, USA).

Cells and Viruses: A549/ACE2/TMPRSS2 cells (provided by M. Saeed, Boston Uni-versity; Boston, MA, USA) were cultured in Growth Media comprised of Dulbecco’s modified Eagle’s medium (DMEM) high glucose supplemented with 10% fetal bovine serum (FBS), 100 IU Streptomycin/Penicillin per mL, 10 mM HEPES, 1X Non-Essential Amino Acids (NEAA), 1X GlutaMAX, 1 mM Sodium Pyruvate, 5 μg/mL Plasmocin, 0.5 μg/mL Puromycin and 0.5 μg/mL Blasticidin. The infection medium was comprised of high-glucose DMEM supplemented with 5% fetal bovine serum (FBS), 100 IU Streptomycin/Penicillin per mL, 10 mM HEPES, 1X Non-Essential Amino Acids (NEAA), 1X GlutaMAX and 1 mM Sodium Pyruvate. SARS-CoV-2 isolate USA-WA1/2020 (NR-52281) was obtained through BEI Resources (Manassas, VA, USA) and propagated in Vero-E6 cells. 

Antiviral evaluation against SARS-CoV-2: 15,000 A549 cells expressing human ACE2 and TMPRSS2 were plated per well into96-well plates and incubated overnight at 37 °C with 5% CO_2_. The next day, the growth medium was removed, and the cells were treated with compound (diluted in infection media) at the indicated concentrations. DMSO concentrations were maintained at 0.5%. Remdesivir (2 μM) was used as a positive control virus inhibitor. After treatment, the cells were inoculated with SARS-CoV-2 USA-WA1/2020 isolate at MOI 0.01. After 2 days, the infection medium was removed, the cells were fixed with 4% PFA, washed three times with PBS and incubated in immunofluorescence (IF) buffer (1X PBS, 0.1% Tween 20) plus 0.3 M Glycine and 1% FBS for 30 min at room temp. Cells were washed three times with IF buffer and incubated with rabbit anti-SARS-CoV-2 nucleoprotein antibody (Sino Biologicals; Houston, TX, USA) in IF buffer + 1% FBS overnight at 4 °C. The next day, the cells were washed with IF buffer (3 times) and incubated with anti-rabbit Alexa 555 (ThermoFisher Scientific; Waltham, MA, USA) antibody for 2 h at room temp. Cells were washed 3 times with IF buffer and 2 times with PBS. Nuclei were counterstained with DAPI (ThermoFisher Scientific; Waltham, MA, USA) to determine the total number of cells per well. Images were acquired in the Cytation One cell imaging multimode reader (BioTek/Agilent; Winooski, VT, USA) using the Gen5 software (BioTek/Agilent; Winooski, VT, USA). The total number of cells and infected cells were quantified in Gen5 software, and the percent of infected cells was calculated by dividing the number of infected cells by total cells in the well and graphed using GraphPad Prism 9 software (GraphPad Software; San Diego, CA, USA).

Antiviral activity assay: The percent (%) of total cells normalized to the DMSO-treated control are shown as white bars. The % infected cells is shown as black bars. Means and standard deviations of triplicate wells are shown. Remdesivir (RDV 2 μM) was used as a positive control and resulted in an almost complete inhibition of infection.

Evaluation of compound cytotoxicity: The cell viability of non-infected cells followed the same experimental outline (cells’ line, cell number, compound concentrations and incubation timing) as their corresponding antiviral assay. Cell viabilities in the presence of drug-candidate inhibitors were determined using the CellTiter 96 Aqueous Non-Radioactive cell proliferation assay (Promega G1111; Madison, WI, USA), a 3-(4,5-dimethylthiazol-2-yl)-5-(3-carboxymethoxyphenyl)-2-(4-sulfophenyl)-2H-tetrazolium (MTS)-based tetrazolium reduction, following the manufacturer’s directions. The data were analyzed to calculate the CC50 values using GraphPad Prism 7 software (GraphPad Software; San Diego, CA, USA).

## Data Availability

Additional data are available in the Supplementary Materials.

## Supplementary Materials

The following supporting information can be downloaded at: https://www.mdpi.com/article/10.3390/molecules28062616/s1. Figure S1: 1H NMR of compound **4A**, Figure S2: 13C NMR of compound **4A**, Figure S3: 19F NMR of compound **4A**, Figure S4: 1H NMR of compound **3A**, Figure S5: 13C NMR of compound **3A**, Figure S6: 19F NMR of compound **3A**, Figure S7: 1H NMR of compound **2A**, Figure S8: 13C NMR of compound **2A**, Figure S9: 19F NMR of compound **2A**, Figure S10: 1H NMR of compound **1A**, Figure S11: 13C NMR of compound **1A**, Figure S12: 19F NMR of compound **1A**, Figure S13: 1H-1H NOESY correlations of compound **1A**.
