# Supplementary material for: Enhanced Remdesivir Analogues to Target SARS-CoV-2
Source: Molecules. 2023 Mar 13;28(6):2616. doi: 10.3390/molecules28062616 (PMC10052049; doi:10.3390/molecules28062616)
Supplement: Supplementary file 1 [file molecules-28-02616-s001.zip › molecules-2237524-supplementary.pdf]

Supplementary Materials

Enhanced Remdesivir Analogues to Target SARS-CoV-2

Ryuichi Majima, Tiffany C. Edwards, Christine D. Dreis,

Robert J. Geraghty* and Laurent F. Bonnac*

Center for Drug Design, College of Pharmacy, University of Minnesota, Minneapolis, MN 55455, USA

Table of Contents:

SI-1 Materials and Methods, Experimental Procedures

SI-2 ^{1}H,^{13}C, ^{19}F, NMR

SI-1 Materials and Methods, Experimental Procedures

All commercial reagents were used as provided unless otherwise indicated. An anhydrous solvent-dispensing system (J.C. Meyer) using two packed columns of neutral alumina was used for drying THF, Et_2O, and CH_2Cl_2, whereas two packed columns of molecular sieves were used to dry DMF. Solvents were dispensed under argon. Flash chromatography was performed with Ultra Pure silica gel (SiliCycle) or with RediSep Rf silica gel columns on a Teledyne ISCO CombiFlash Rf system using the solvents as indicated. All reactions were performed under a dry atmosphere of argon unless otherwise specified. Indicated reaction temperatures refer to the reaction bath, while room temperature (rt) is noted as 25°C. Commercial grade reagents and anhydrous solvents were used as received from vendors, and no attempts were made to purify or dry these components further. Removal of solvents under reduced pressure was accomplished with a Buchi rotary evaporator at approximately 28 mmHg pressure using a Teflon-linked KNF vacuum pump. Thin layer chromatography was performed using either 1 in. × 3 in. Anal Tech No. 02521 or Merck 60 F254 silica gel plates with fluorescent indicator using appropriate solvent mixtures. Visualization of TLC plates was made by observation with either short wave UV light (254 nm lamp) or 10% sulfuric acid in ethanol. Nuclear magnetic resonance spectra were recorded on a Varian 600 MHz or Bruker 400 spectrometer with Me4Si or signals from residual solvent as the internal standard for 1H. Chemical shifts are reported in ppm, and signals are described as s (singlet), d (doublet), t (triplet), q (quartet), m (multiplet), br s (broad singlet), and dd (double doublet). Values given for coupling constants are of first order. Mass spectroscopic analyses were performed either using positive mode electron spray ionization (ESI) on a Varian ProStar LC-MS with a 1200L quadrupole mass spectrometer or using positive mode atmospheric pressure chemical ionization (APCI) on a Shimadzu LC-MS system. High performance

liquid chromatography (HPLC) purity analysis was conducted using a Varian Pro Star HPLC system with a binary solvent system A and B using a gradient elution [A, H2O with 0.1% trifluoroacetic acid (TFA); B, CH_3CN with 0.1% TFA] and flow rate = 1 mL/min, with UV detection at 254 nm. All final compounds were purified to ≥95% purity, and these purity levels were measured by a Varian Pro Star HPLC system. Three different Varian Pro Star HPLC methods were used to establish compound purity. HPLC Method A: Phenomenex Luna C18(2) column (4.6 mm × 250 mm); mobile phase, A = H2O with 0.1% TFA and B = CH_3CN with 0.1% TFA; gradient 10−95 % B (0.0−10 min; hold for 6 min); UV detection at 254 nm. HPLC Method B: SunFire C18 column (4.6 mm × 250 mm); mobile phase, A = H2O with 0.1% TFA and B = CH_3CN with 0.1% TFA; gradient 10−100 % B (0.0−20 min; hold for 5 min); UV detection at 254 nm. HPLC Method C: SunFire C18 column (4.6 mm × 250 mm); mobile phase, A = H_2O with 0.1% TFA and B = CH_3CN with 0.1% TFA; gradient 0−100 % B (0.0−15 min; hold for 5 min)); UV detection at 254 nm. IR measurements were performed with JASCO (FT/IR-4100) Fourier Transform Infrared Spectrometer, neat film analysis.

Compound 4A

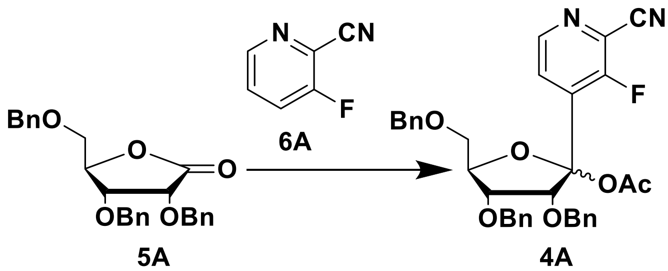

5A 4A

3-fluoro-2-pyridinecarbonitrile (6A) (1.1eq, 3.2g, 26.28mmol) and 2,3,5-tri-O-benzylribonolactone (5A) (1eq, 10g, 23.89mmol) are dried under vacuum overnight in separate flasks. (6A) is then dissolved in dry THF (5mL/mmol, 130mL) and cooled to -78°C with a dry ice/acetone bath for 20 minutes then Lithium Diisopropyl Amide LDA solution (2M THF) (1.1eq, 26.28mmol, 13.14mL) is added dropwise (2-3minutes) and

stirred at -78°C for 30 minutes. A solution of (5A) in dry THF (4mL/mmol, 100mL), precooled to -78°C, is then cannulated to the (6A/LDA) mixture and stirred at -78°C for 1.5 hour. The mixture is quenched with saturated ammonium chloride solution (200mL) and extracted with Ether (3x200mL). The organic phases are combined and washed with water, then concentrated to dryness, co-evaporated with methanol, then toluene. The mixture is dissolved in dry THF (5mL/mmol, 130mL). Lithium bis(trimethylsilyl)amide (LiHMDS) (1M in THF) (1.5eq, 35.83mmol, 35.83mL) is added at room temperature and stirred for 10 minutes. Acetic anhydride (1.5eq, 35.83mmol, 6.7mL) is added and stirred for 20 minutes. An additional 0.75eq of LiHMDS and 0.75eq of acetic anhydride are added to the reaction mixture and stirred for 20 minutes. The reaction mixture is dissolved into 400mL of toluene and washed with saturated $NaHCO_3$ solution (4x100mL). The organic phase is concentrated to a minimum, diluted with 4mL of DCM and purified on silica gel chromatography Hexane, AcOEt (0%, 10% then 20%) to obtain the desired compound (4A) 6.54g, yield 47% as a light yellow thick oil Rf= 0.1 (hexane, ethyl acetate 20%), or Rf= 0.5 (hexane, ethyl acetate 30%).

^{1}H NMR (400 MHz, $CDCl_3$) δ 8.04 (d, J = 4.92 Hz, 1H), 7.57(t, J = 5.04 Hz, 1H), 7.4-7.0 (m, 15H), 4.61 (m, 2H), 4.42 (m, 2H), 3.96 (m, 1H), 3.73 (m, 2H), 3.46 (m, 2H), 2.09 (s, 3H).^{13}C NMR (100 MHz, $CDCl_3$) δ 169.4, 158.6-155.9, 146.8, 137.7, 137.6, 137.5, 137.4, 136.3, 128.6, 128.4, 128.3, 128.2, 128.1, 128.0, 127.9, 127.9, 127.8, 127.7, 127.6, 126.9, 126.4, 123.2, 123.1, 123.1, 113.1, 102.9, 84.2, 83.5, 75.1, 74.1, 73.7, 73.6, 69.2, 21.4. ^{19}F NMR (376 MHz, $CDCl_3$) δ -119.7. HRMS (ESI$^+$): m/z calculated for $C_{34}H_{31}FN_2NaO_6^+$ $[M+Na]^+$ 605.2058, found 605.2058. Neat film, IR 2344 cm^{-1} (CN, m, br), 1741 cm^{-1} (CO, s, br).

Compound 3A

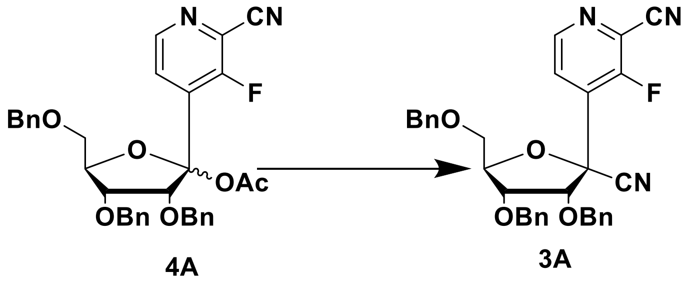

Compound (4A) (1eq, 2.2g, 3.77mmol) is dissolved in DCM (10mL/mmol, 40mL) under argon atmosphere, TMSCN (use with caution! 3.8eq, 14.20mmol, 1.8mL) is added and the mixture is cooled -78°C. After 15 minutes at -78°C, Boron trifluoride diethyl etherate ($BF_3.Et_2O$) is added dropwise to the mixture, then stirred at -78°C for 2 hours and 0°C for 1 hour. The mixture is diluted into 100mL of DCM and quenched with 100mL of NaHCO3 sat and stirred at room temperature for 20 minutes. The organic phase is separated. The aqueous phase is extracted with DCM (3x100mL). The aqueous phase is poured into 200mL of $KMnO_4$ solution to oxidize the remaining reagent. The organic phases are combined, 35mL of silica is added and the mixture is concentrated for solid deposit silica gel chromatography Hexane, AcOEt (0 to 20%), Rf= 0.25 (hexane,ethyl acetate 20%), to obtain 1.05g (51%) of a colorless thick oil which solidifies to a white paste overtime.

^{1}H NMR (400 MHz, $CDCl_3$) δ 8.10 (d, J = 4.92 Hz, 1H), 7.79(t, J = 5.04 Hz, 1H), 7.4-7.0 (m, 15H), 4.69 (s, 1H), 4.42 (m, 6H), 4.07 (m, 1H), 3.89 (m, 1H), 3.66 (dd, 2H). ^{13}C NMR (100 MHz, $CDCl_3$) δ 158.9-156.2, 147.2, 137.6, 137.3, 137.0, 136.8, 136.0, 134.8, 134.7, 129.7, 129.2, 129.0, 128.7, 128.6, 128.4, 128.2, 128.0, 127.8, 126.6, 126.3, 123.6, 123.4, 115.3, 112.4, 83.0, 82.7, 78.1, 75.3, 74.9, 73.8, 73.6, 72.8, 67.9. ^{19}F NMR (376 MHz, $CDCl_3$) δ -116.2 HRMS (ESI$^+$): m/z calculated for $C_{33}H_{29}FN_3O_4^+$ $[M+H]^+$ 550.2137, found 550.2140. Neat film, IR 2336cm^{-1} (CN, m, br).

Compound 2A

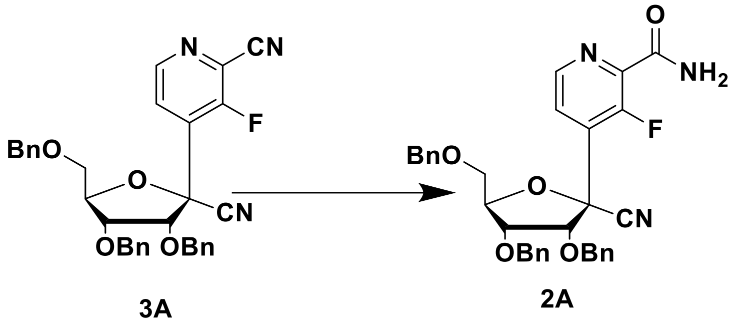

Compound 3A (1eq, 0.5g, 0.9mmol) is introduced in a flask then 20mL of acetone, 20mL of methanol and 20 mL of water then sodium perborate tetrahydrate (3eq, 0.42g, 2.73mmol) is added. The mixture is stirred at 50°C for 3 hours. The mixture is concentrated to dryness and purified on silica gel chromatography (Hexane, Ethyl acetate 0 to 30%) colorless oil, 0.2g 40% yield. Rf= 0.25 (hexane/ethyl acetate, 1/1). ^{1}H NMR (400 MHz, $CDCl_3$) δ 8.10 (d, J = 4.92 Hz, 1H), 7.79(t, J = 5.04 Hz, 1H), 7.4-7.0 (m, 15H), 4.69 (s, 1H), 4.42 (m, 6H), 4.07 (m, 1H), 3.89 (m, 1H), 3.66 (dd, 2H). ^{13}C NMR (100 MHz, $CDCl_3$) δ 164.1-164.0, 157.0-154.3, 144.4, 144.3, 137.7, 137.6, 137.6, 137.1, 136.5, 136.0, 135.9, 128.5, 128.4, 128.4, 128.3, 128.2, 128.0, 127.9, 127.8, 127.8, 127.6, 127.5, 126.1, 115.6, 82.4, 81.5, 79.0, 75.0, 74.2, 73.4, 72.6, 67.3. ^{19}F NMR (376 MHz, $CDCl_3$) δ -120.2 HRMS (ESI$^+$): m/z calculated for $C_{33}H_{31}FN_3O_5^+$ $[M+H]^+$ 568.2242, found 568.2248. Neat film, IR 2357cm^{-1} (CN, m, br), 1650 cm^{-1} (CO, w).

Compound 1A

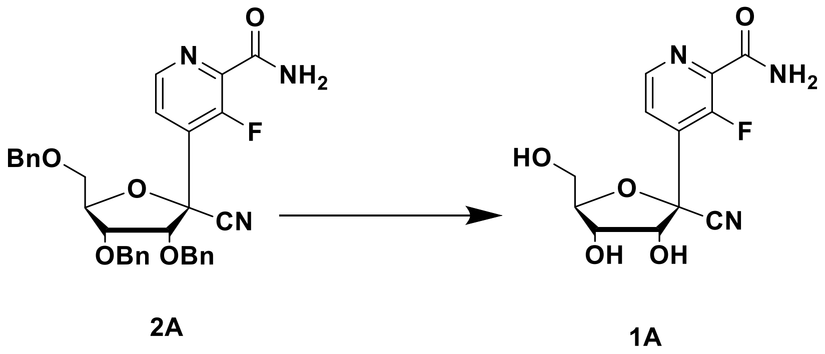

Compound 2A (1eq, 0.150g, 0.26mmol) and benzamide (1eq, 32mg, 0.26mmol) are dissolved into DCM (2mL/mmo, 10mL) and cooled to -78°C and stirred for 5 minutes. Boron tribromide 1N in DCM (4eq, 1.04mL, 1.04mmol) is added dropwise and the mixture is left to warm up to room temperature overnight. The mixture is quenched with 40mL of (Et_2O, MeOH 3/1) and stirred at room temperature for 10 minutes then concentrated to dryness and purified on silica gel chromatography (AcOEt, MeOH 0 to 20%), Rf,= 0.65 (ethyl acetate, methanol 25%) colorless paste yield 20%.

^{1}H NMR (400 MHz, D_2O) δ 8.52 (d, J = 4.92 Hz, 1H), 8.03(t, J = 5.04 Hz, 1H), 4.34 (d, 1H), 4.26 (m, 1H), 4.04 (m, 1H), 3.87 (dd, 2H). ^{13}C NMR (100 MHz, D_2O) δ 165.85-165.81, 156.7-154.0, 144.9-144.8, 139.1-139.0, 136.4-136.3, 124.6, 116.0, 85.1, 80.1-80.0, 77.9, 69.5, 60.4. ^{19}F NMR (376 MHz, $CDCl_3$) δ -122.0 HRMS (ESI$^+$): m/z calculated for $C_{12}H_{13}FN_3O_5^+$ [M+H]$^+$298.0834, found 298.0828. Neat film, IR 2347cm^{-1} (CN, m, br), 1548cm^{-1} (CO, w).

SI-2 1H,13C, 19F, NMR

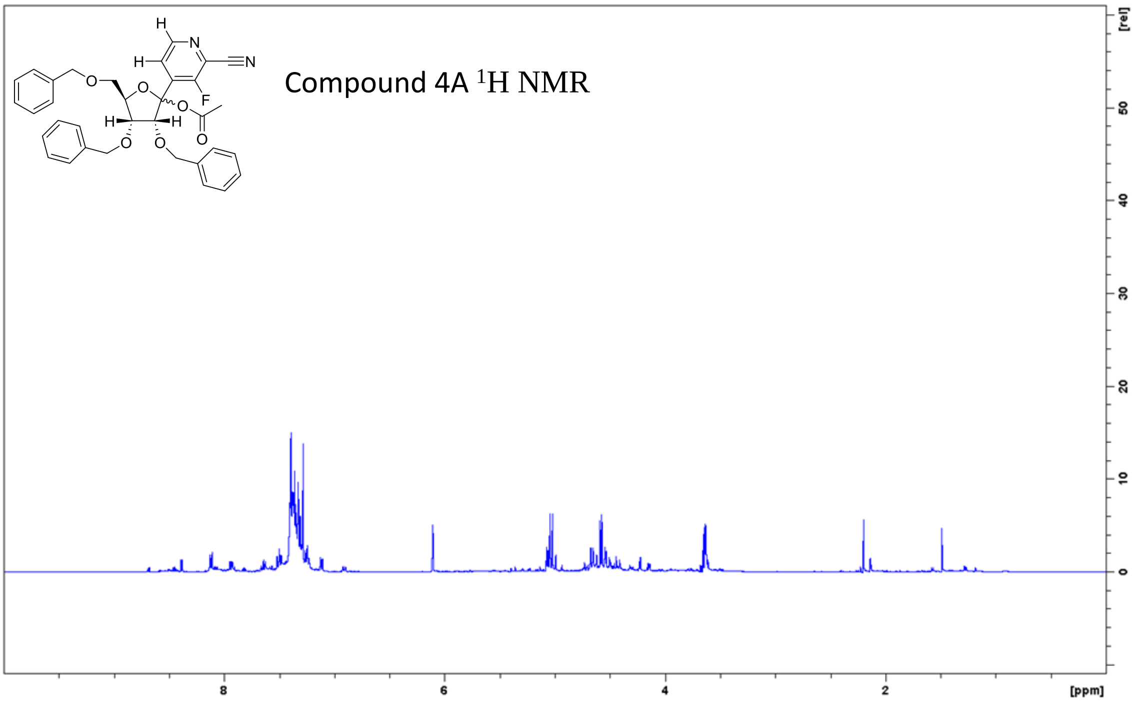

Figure S1: ^{1}H NMR of compound 4A

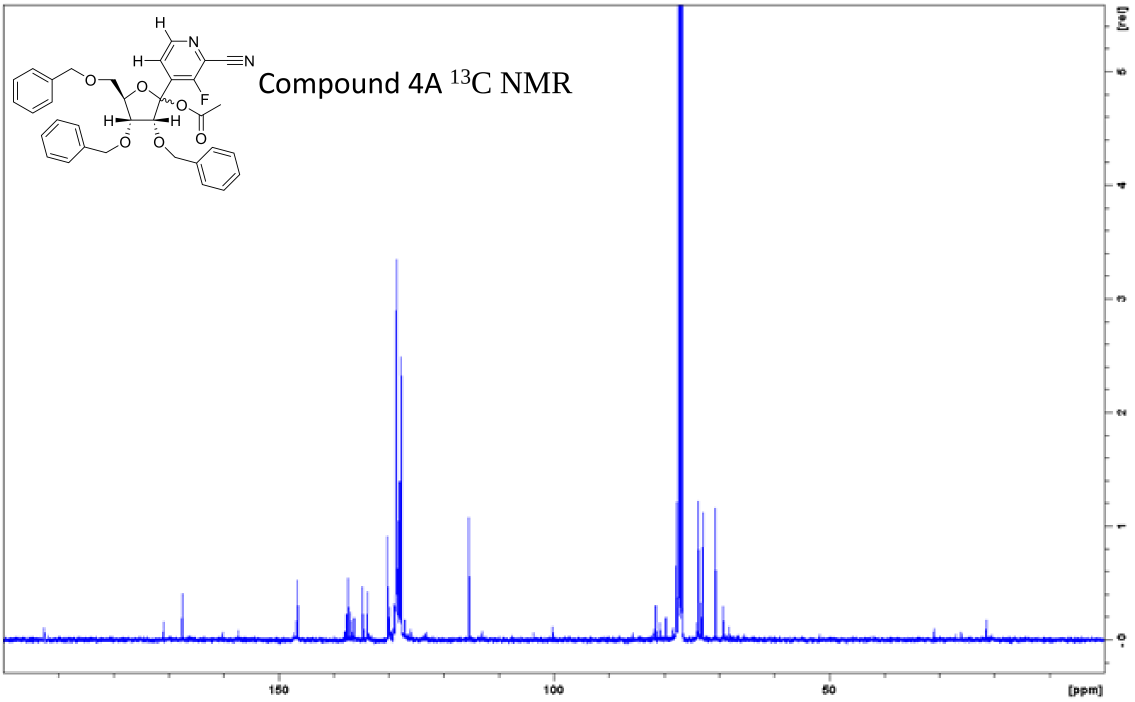

Figure S2: ^{13}C NMR of compound 4A

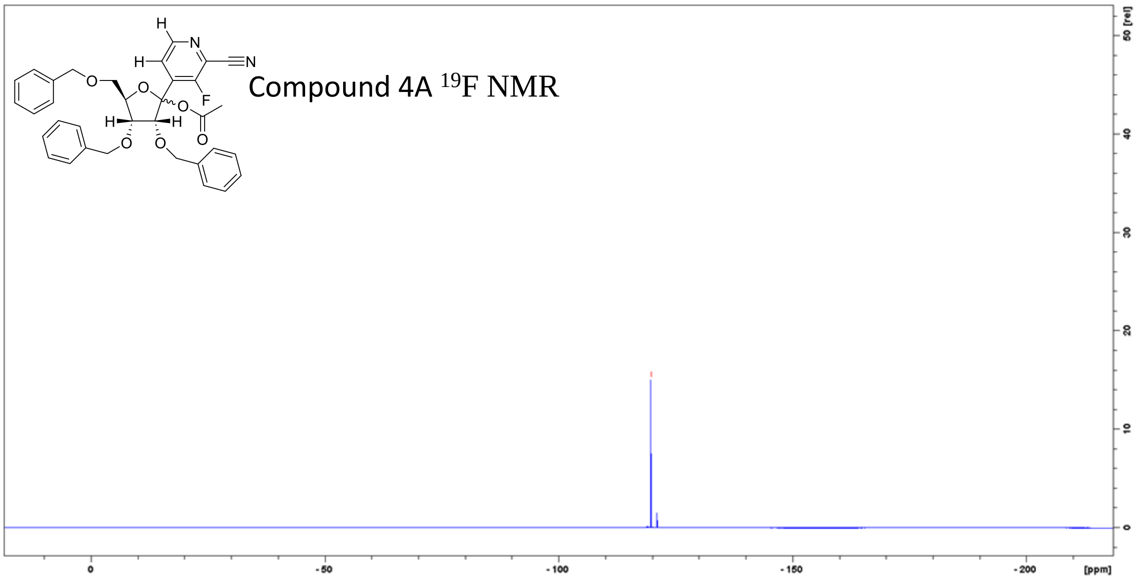

Figure S3: ^{19}F NMR of compound 4A

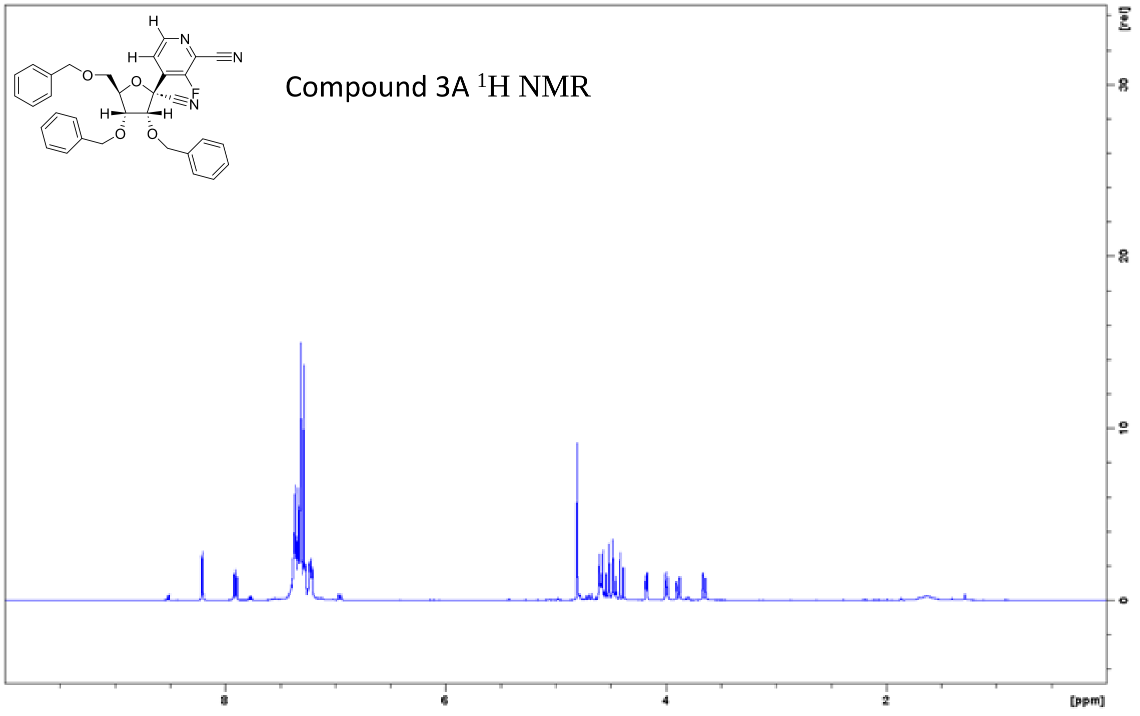

Figure S4: ^{1}H NMR of compound 3A

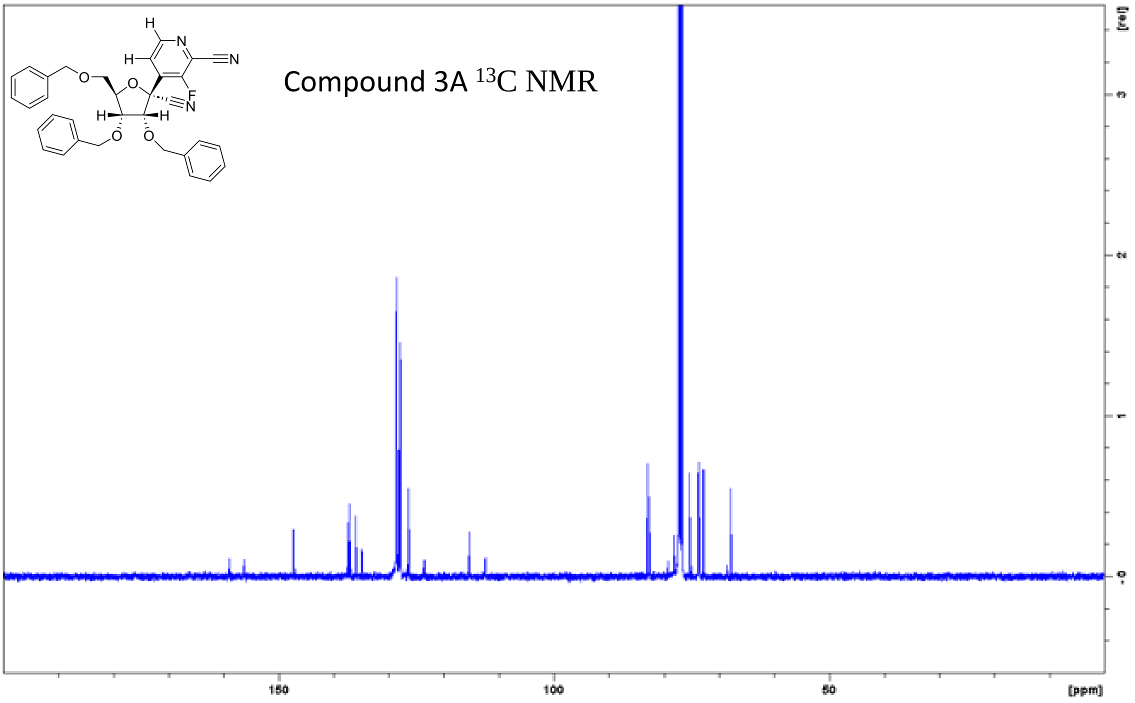

Figure S5: ^{13}C NMR of compound 3A

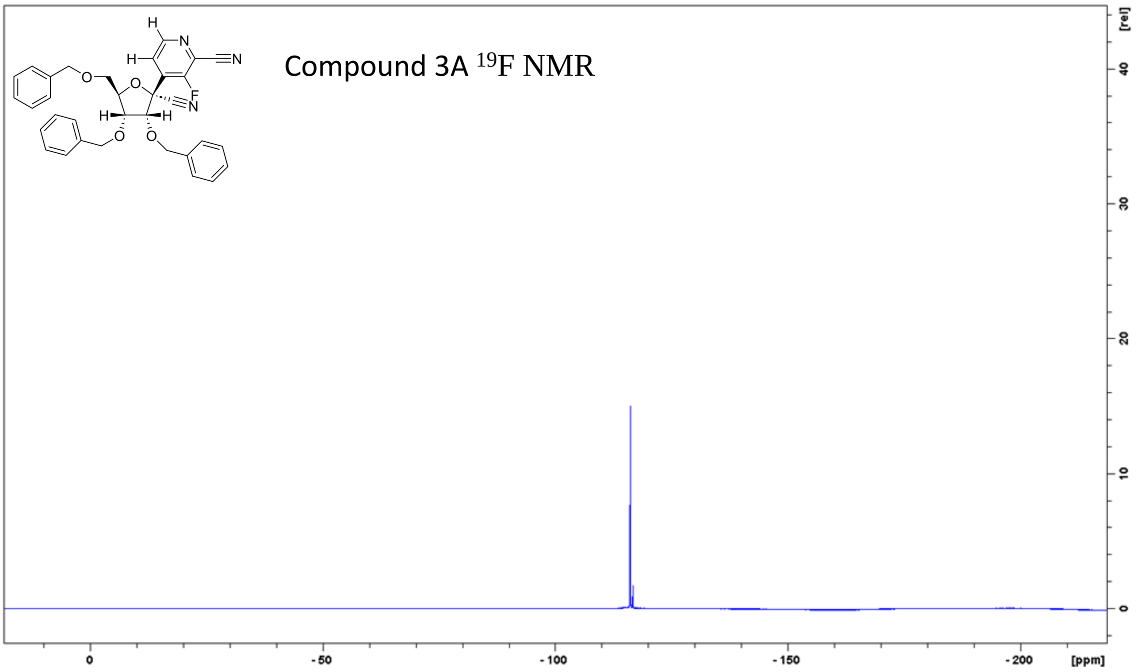

Figure S6: ^{19}F NMR of compound 3A

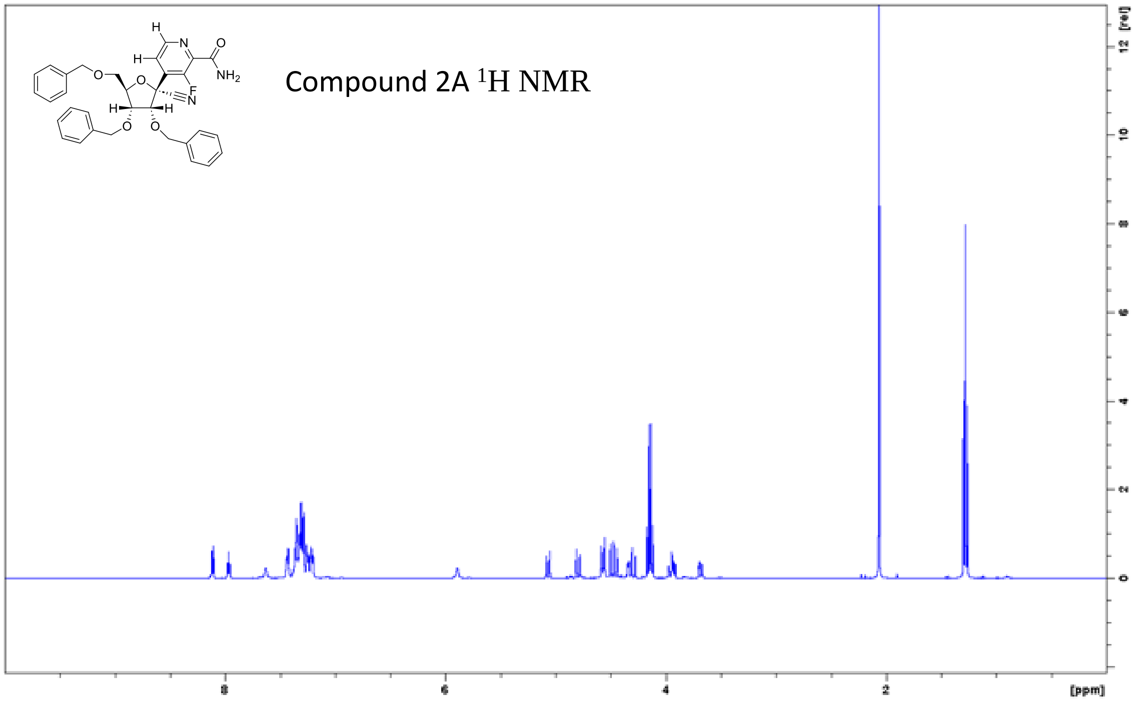

Figure S7: ^{1}H NMR of compound 2A

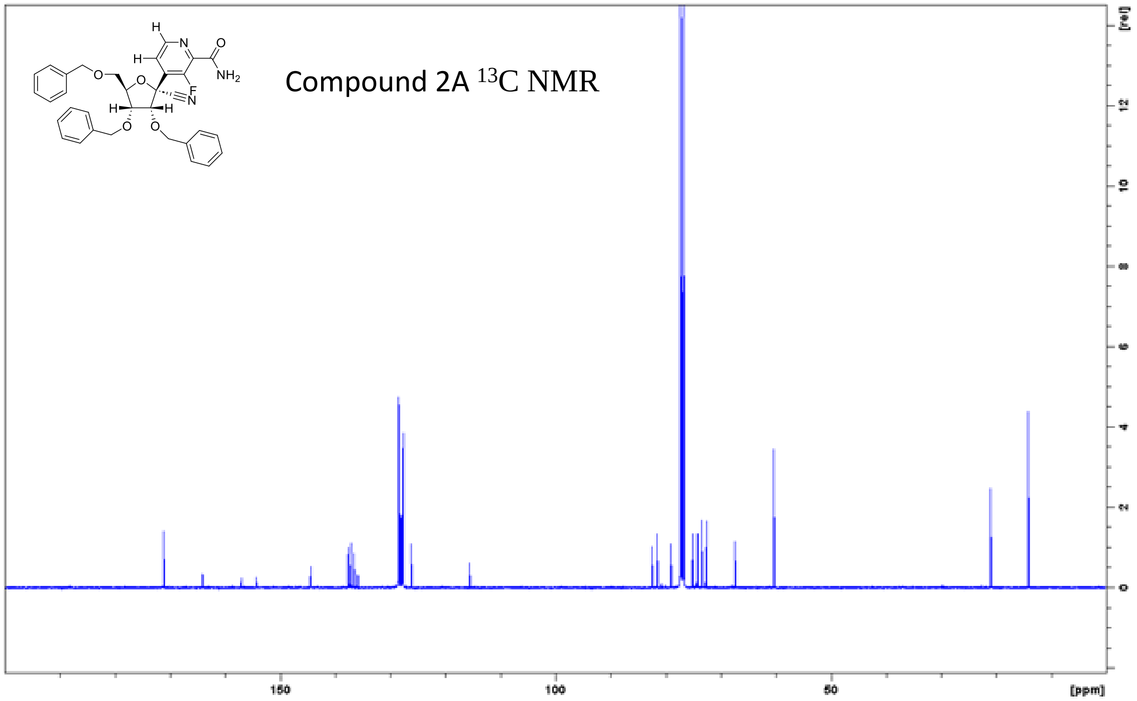

Figure S8: ^{13}C NMR of compound 2A

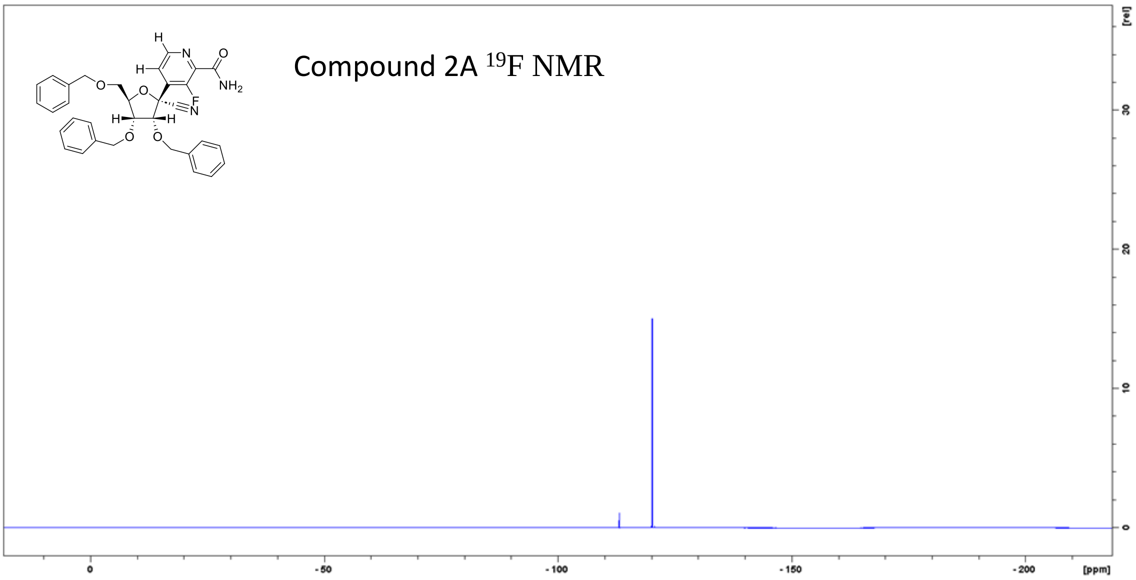

Figure S9: ^{19}F NMR of compound 2A

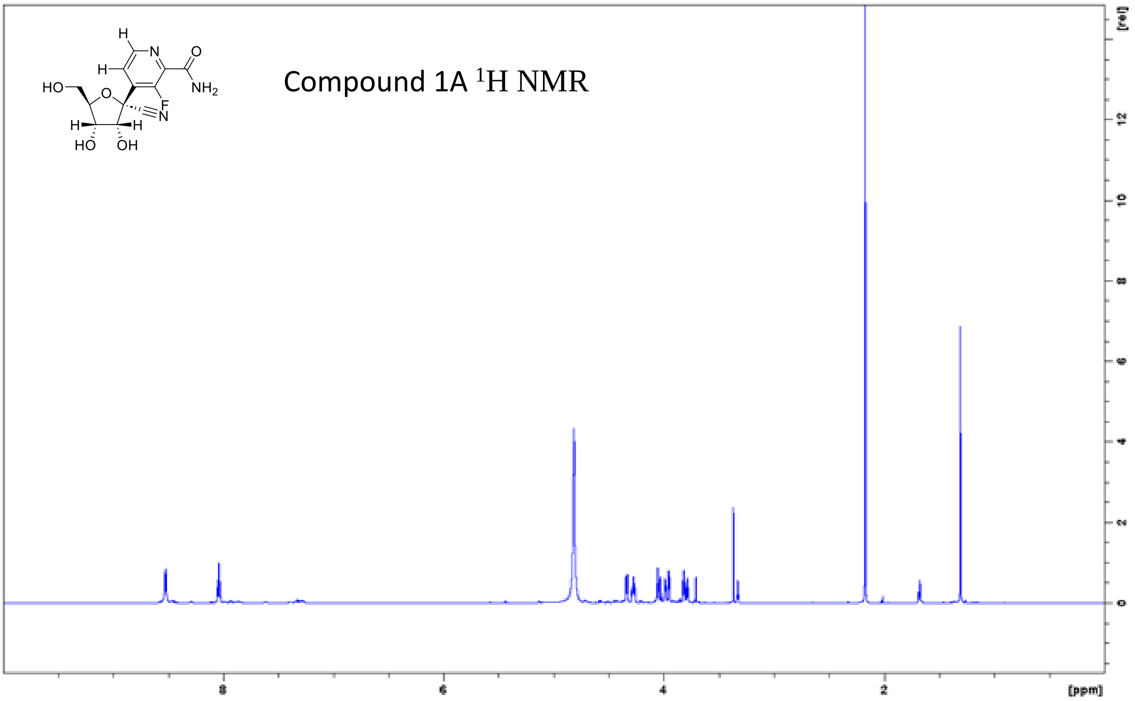

Figure S10: ^{1}H NMR of compound 1A

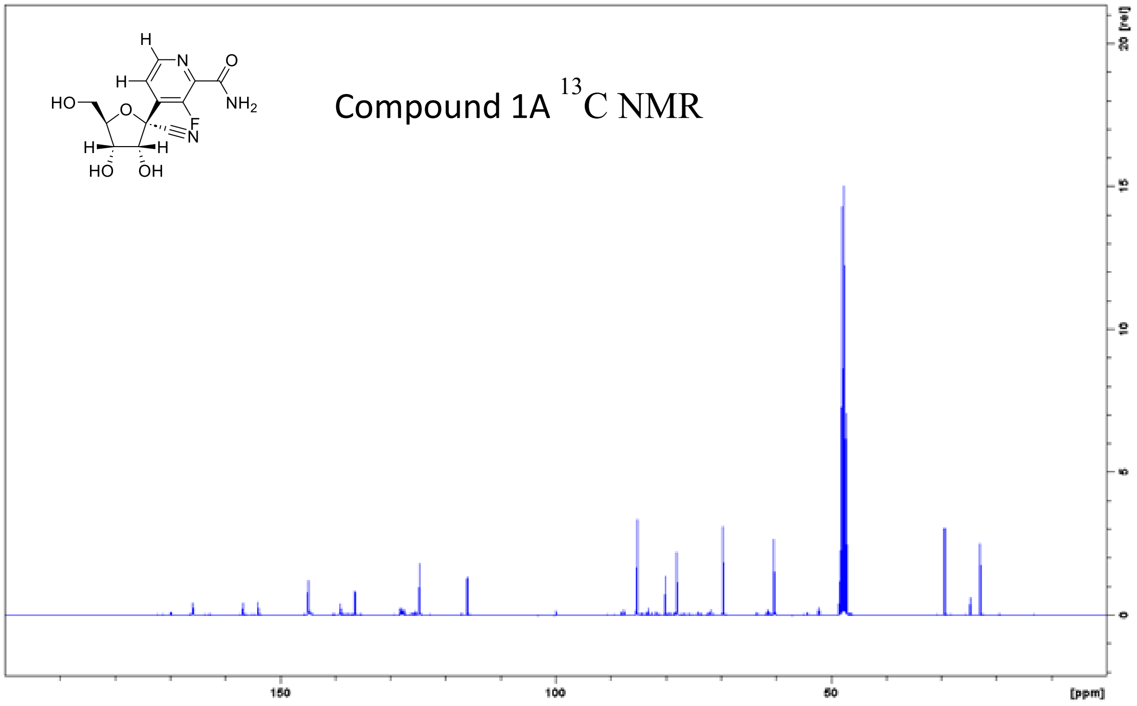

Figure S11: ^{13}C NMR of compound 1A

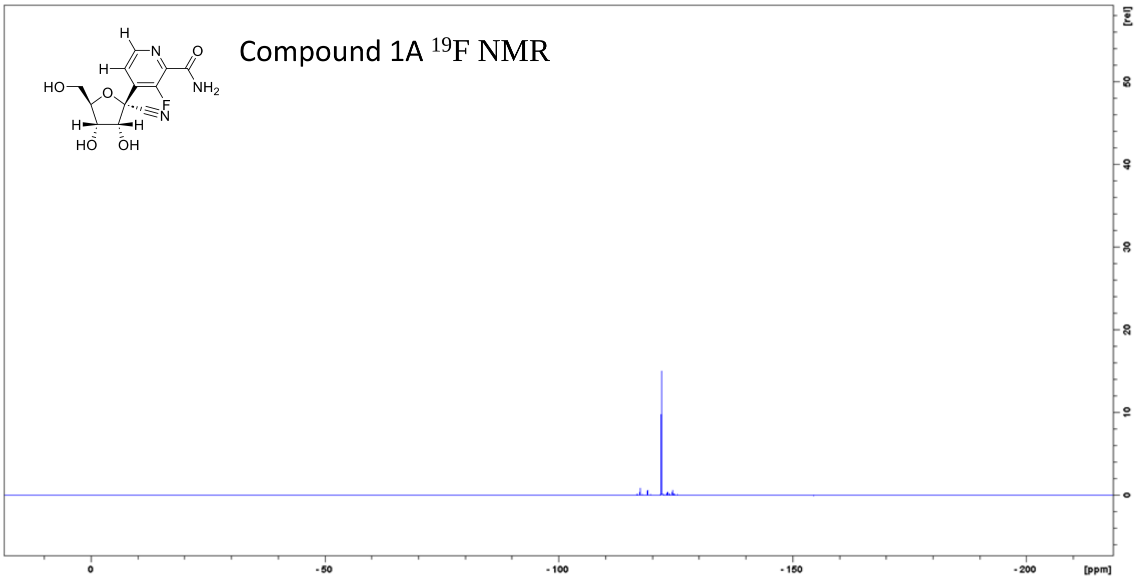

Figure S12: ^{19}F NMR of compound 1A

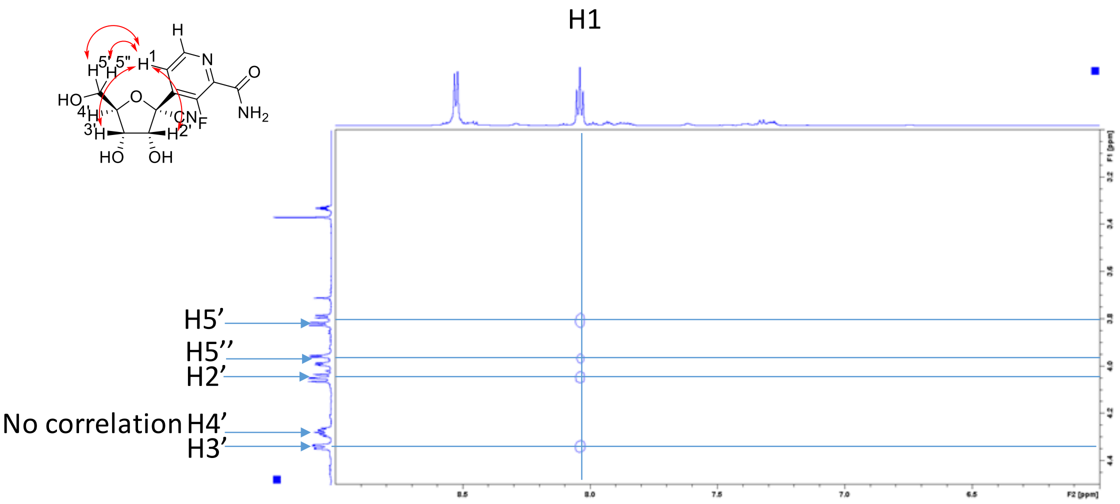

Figure S13: ^{1}H-^{1}H NOESY correlations of compound 1A
